# Supplementary material for: In situ observation of oscillatory redox dynamics of copper
Source: Nat Commun. 2020 Jul 16;11:3554. doi: 10.1038/s41467-020-17346-7 (PMC7366672; doi:10.1038/s41467-020-17346-7)
Supplement: Supplementary file 1 — Supplementary Information [file 41467_2020_17346_MOESM1_ESM.docx]

## Supplementary information

***In situ* observation of oscillatory redox dynamics of copper**

Cao *et al.*


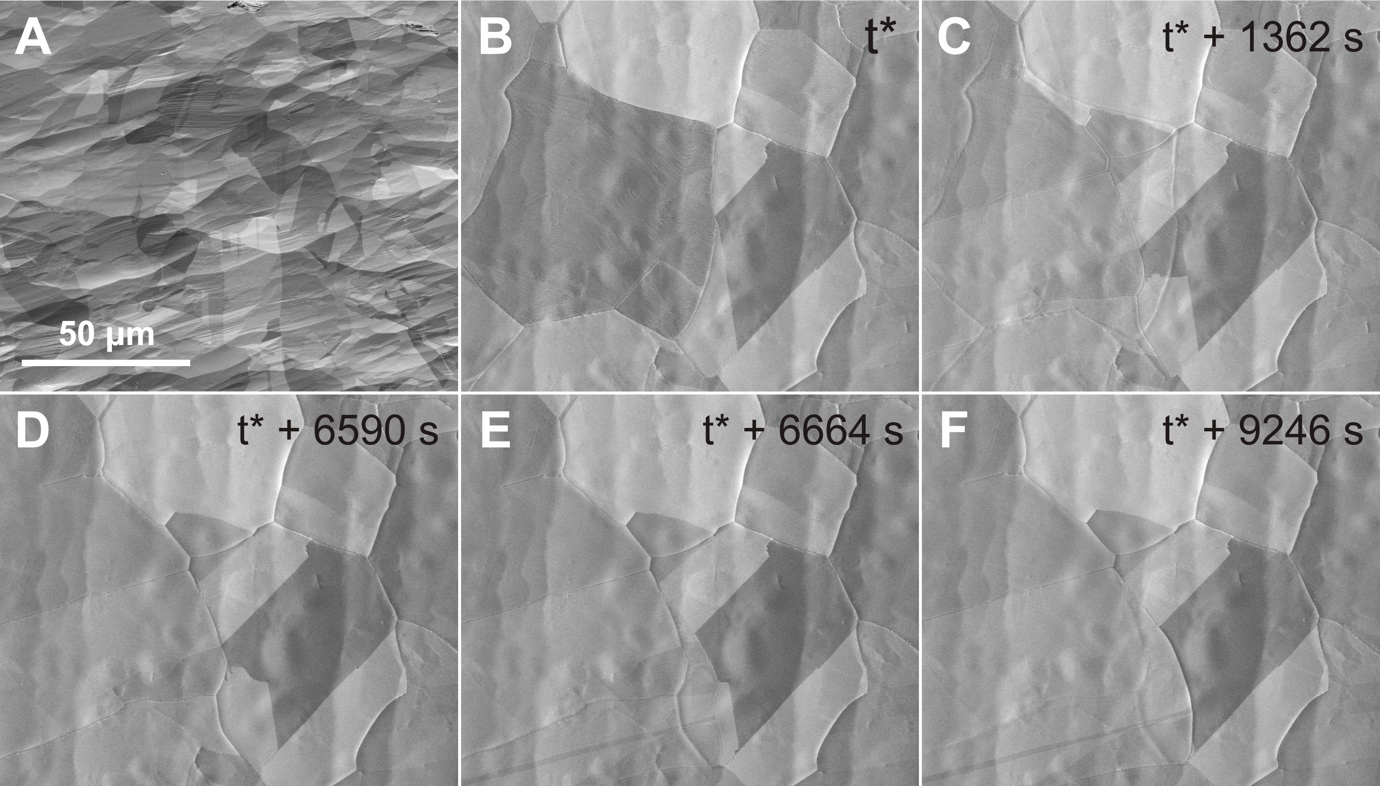


**Supplementary Figure 1:** A) shows the morphology of a copper foil after ion milling of the surface. In B-F), grain growth and migration during annealing in 20 Pa hydrogen at 700 °C is shown. The contrast between differently oriented copper grains is indicative for a clean surface with a high degree of surface reconstruction.


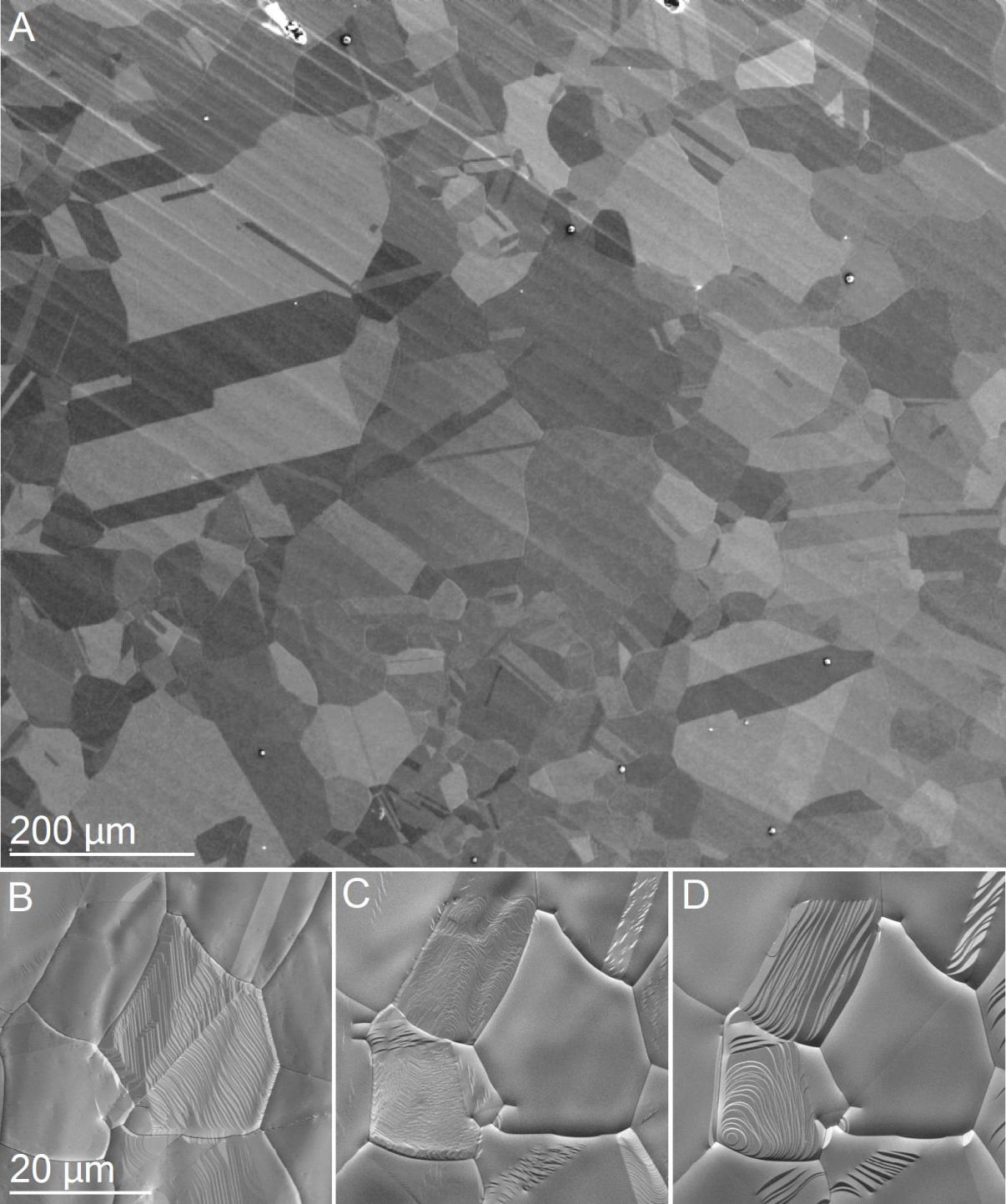


**Supplementary Figure 2**: SEM of oxygen induced surface reconstruction. **A:** Well annealed and reduced surfaces Cu surfaces show a flat morphology. **B-D:** images recorded during H_2_ annealing at 700 °C. The central grain is still showing a faceted surface in **B** due to insufficient reduction. Complete surface flattening in pure hydrogen is only reached after prolonged annealing at high temperature (700 °C). This confirms that stepped surfaces formed due to oxygen induced reconstructions are very stable. **C**: After closing the H_2_ flow, evacuation of the chamber to 5×10^-5^ Pa and introducing 5×10^-3^ Pa O_2_, the surface of the central grain flattened, while other grains developed facets. **D**: Upon further increase of the O_2_ pressure to 1×10^-2^ Pa, the surface of some grains shows a different type of reconstruction.


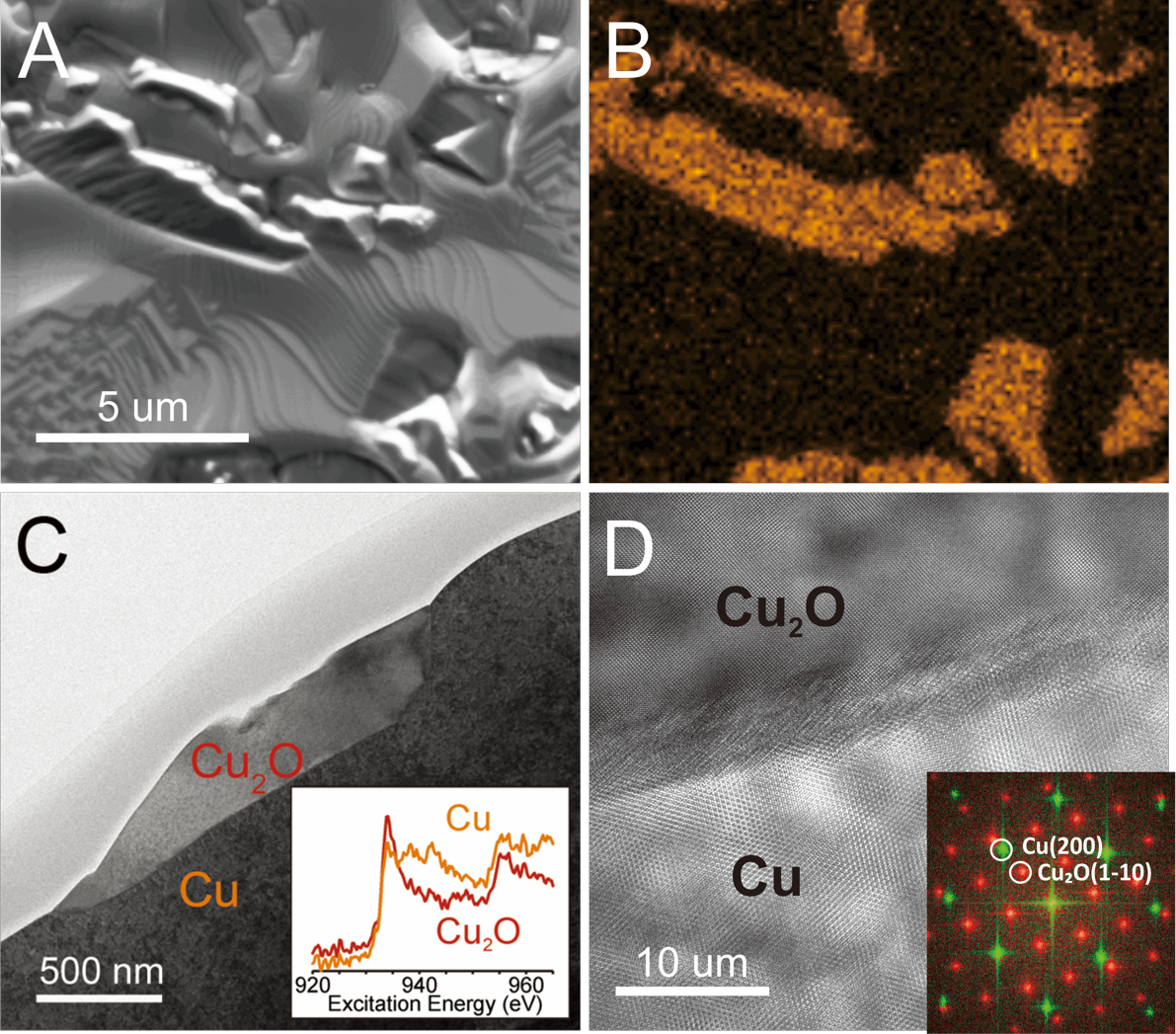


**Supplementary Figure 3: A:** SEM image recorded after quenching the redox-dynamics observed in region B of the dynamic phase diagram. **B:** corresponding energy dispersive X-ray (EDX) map showing that oxide islands with distinct morphology coexist besides metallic copper. EDX is not sensitive enough to detect differences in oxygen concentration between metallic domains that show different surface structure.


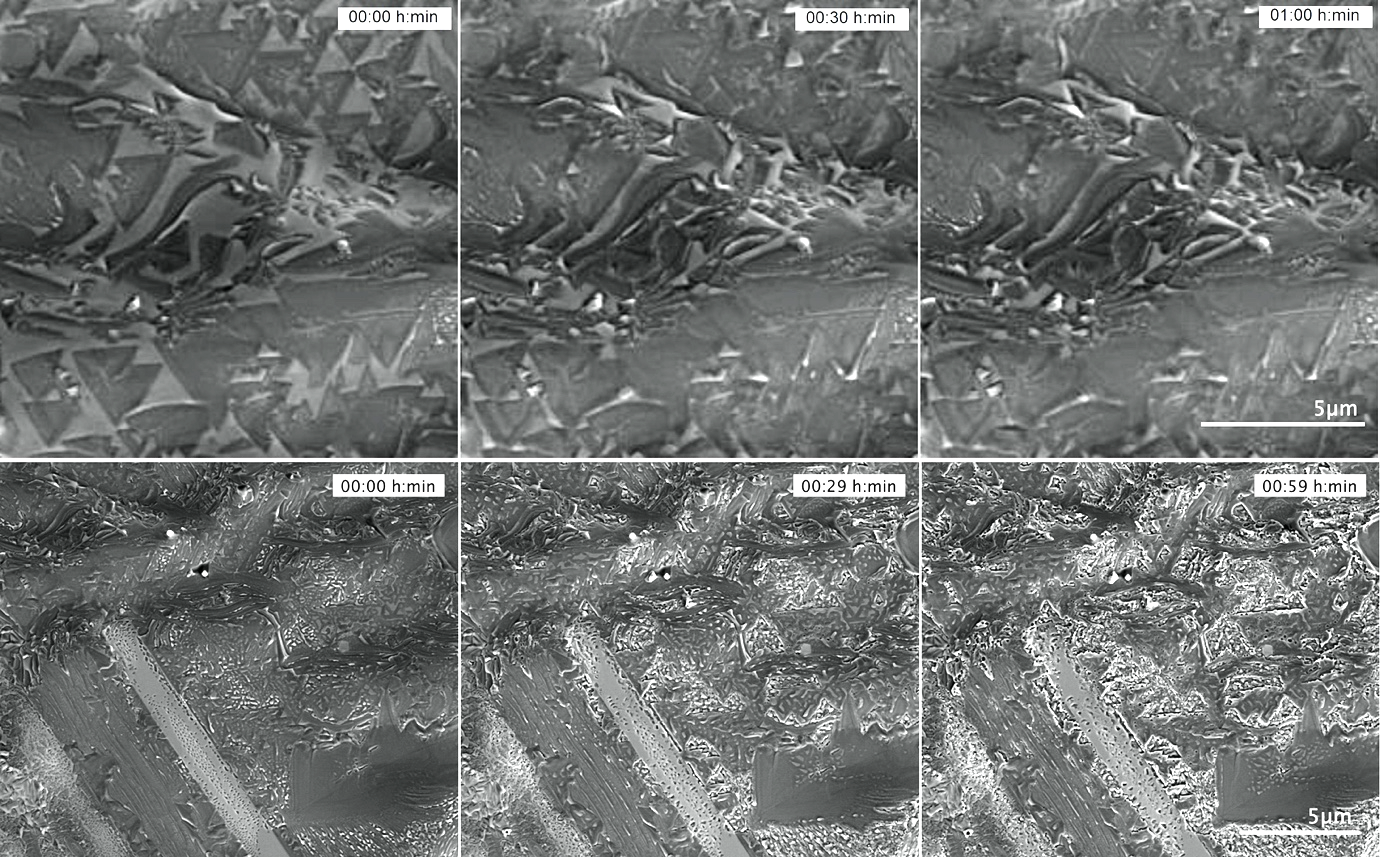


**Supplementary Figure 4:** Morphological changes observed during surface oxidation in an atmosphere containing 94% H_2_ and 6% O_2_. Top row: slow surface oxidation at 300 °C, bottom row: besides slow oxide growth, also the collapse of oxide structures due to reduction can be observed at 400 °C.

**Supplementary Figure 5*.*** MS data recorded during the redox-reaction of H_2_ and O_2_ on copper as a function of temperature. H_2_/O_2_ ratio was 10:1.


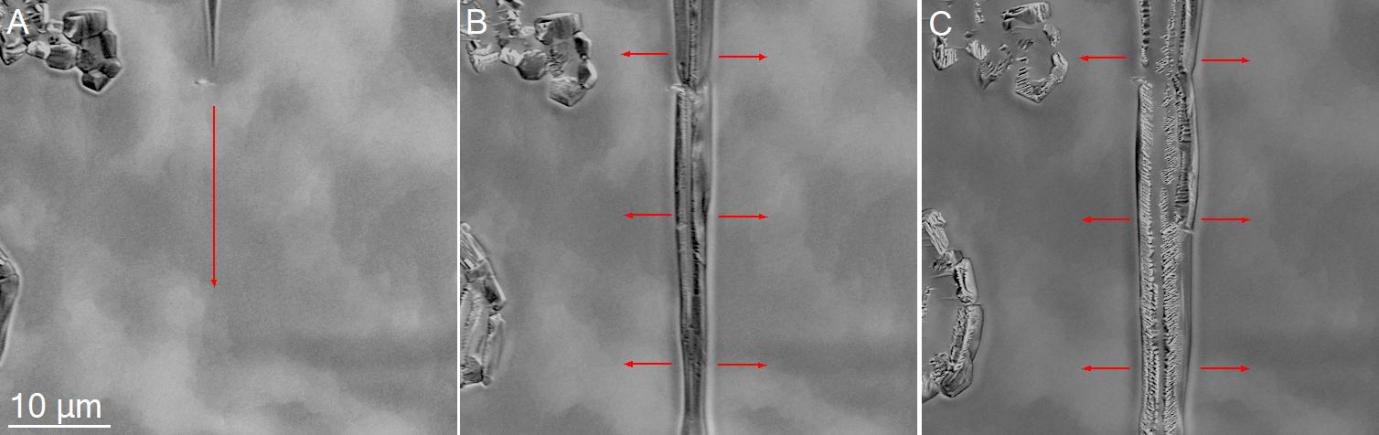


**Supplementary Figure 6**: Oxide island growing on a Cu [110] single crystal in the redox regime. Fast growth of the oxide island along the Cu [111] direction (see FIB-TEM analysis in main manuscript). Sideward growth is accompanied with the formation of a bow-wave in the metallic copper.


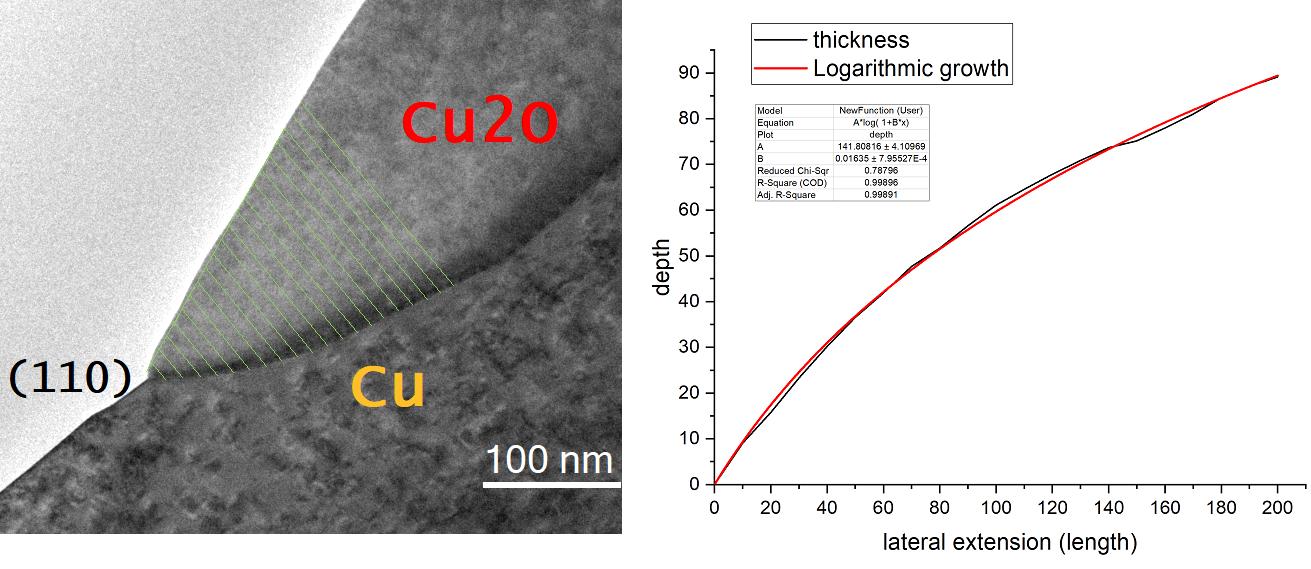


**Supplementary Figure 7:** *A*) Logarithmic growth of oxide thickness with time (time axis is here expressed as distance from the growth-front).


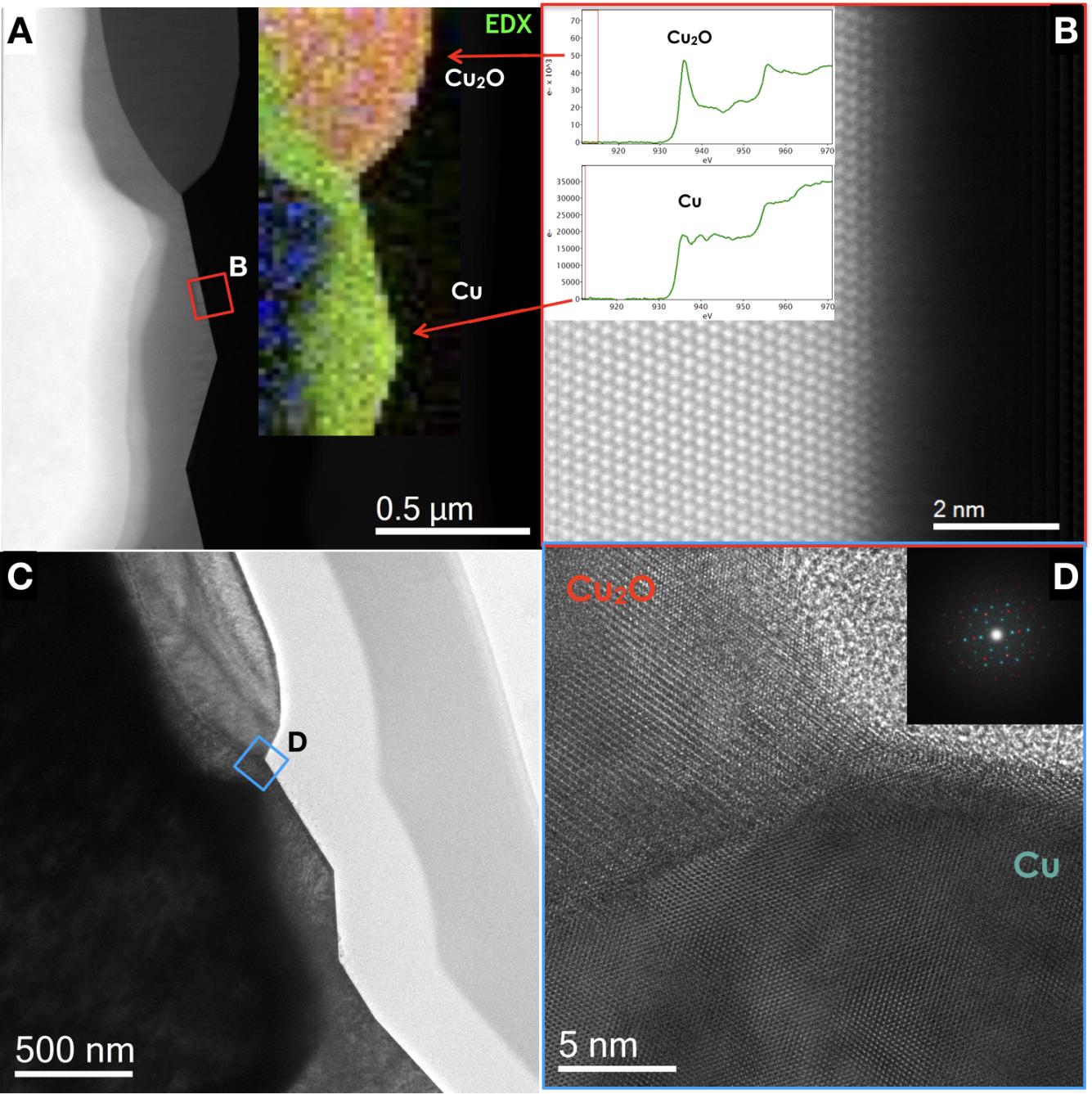


**Supplementary Figure 8:** Analytical TEM observation of oxide growth on metallic copper. The FIB sample was prepared after quenching oxide growth in pure oxygen at a pressure of 8*10^-3^ Pa at 700 °C. **A:** Overview high-angle annular dark-field (HAADF) STEM image. The oxide is identified by EDX and EELS as Cu_2_O. **B:** The metallic Cu facets appear atomically flat in HAADF-STEM imaging. Atomistic details of the topmost atomic layer and possible hints about an oxygen induce surface reconstruction are hidden due to the fading contrast and relatively high thickness of the FIB lamella. **C:** Oxide island with facetted bow-wave. **D:** high-resolution TEM image showing an inclined Cu/Cu_2_O interface and order-order transition between oxide and metallic copper.

*
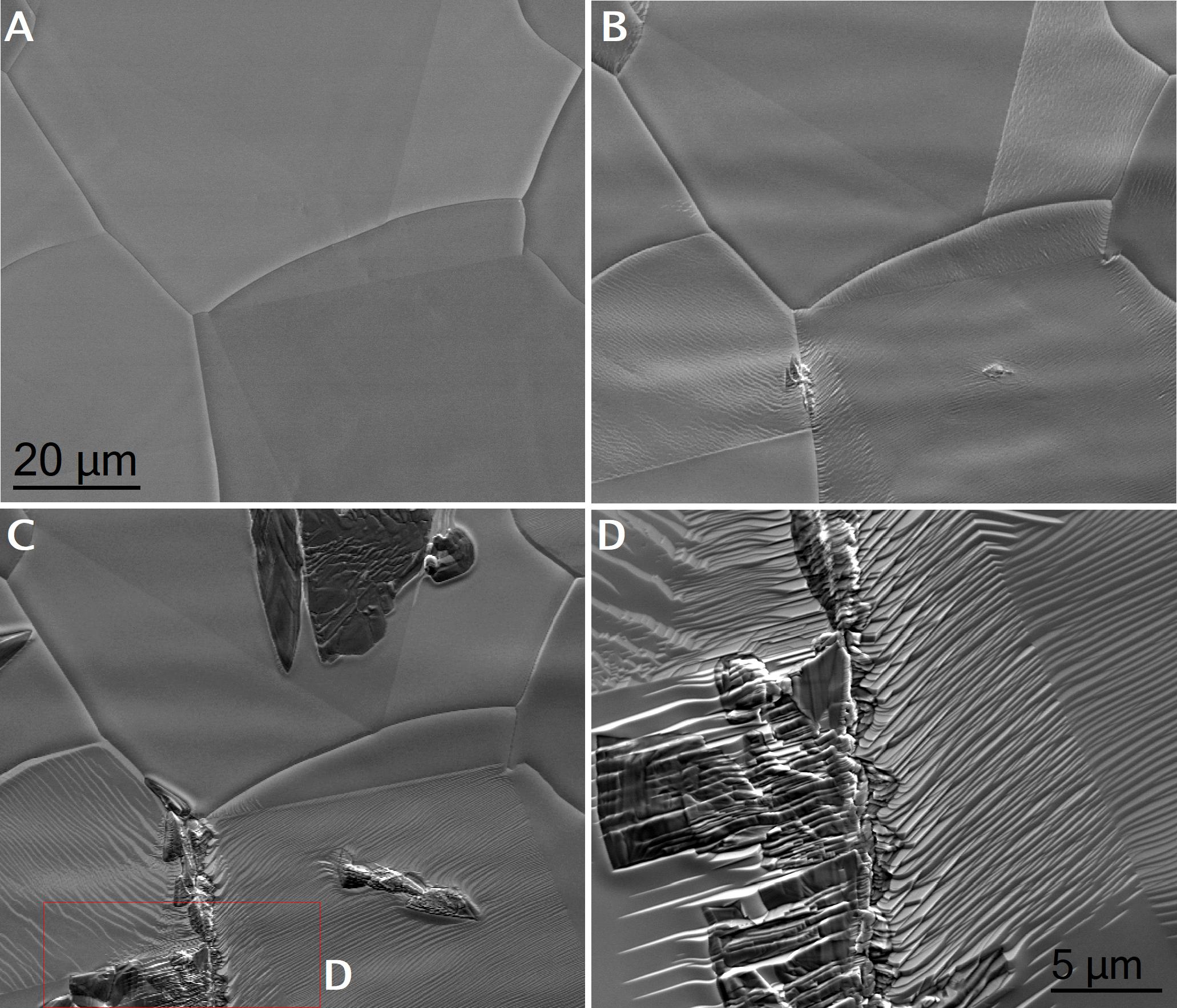
*

**Supplementary Figure 9:** Cu oxidation in pure oxygen: **A:** flat Cu surface after H_2_ annealing. **B:** oxide growth after nucleation at a grain boundary. **C:** oxide islands growing on flat and stepped Cu surface. **D:** magnified region (indicated in **C**), showing growth of Cu_2_O on stepped Cu surface. Growth conditions: 8*10^-3^ Pa O_2_, 700 °C.


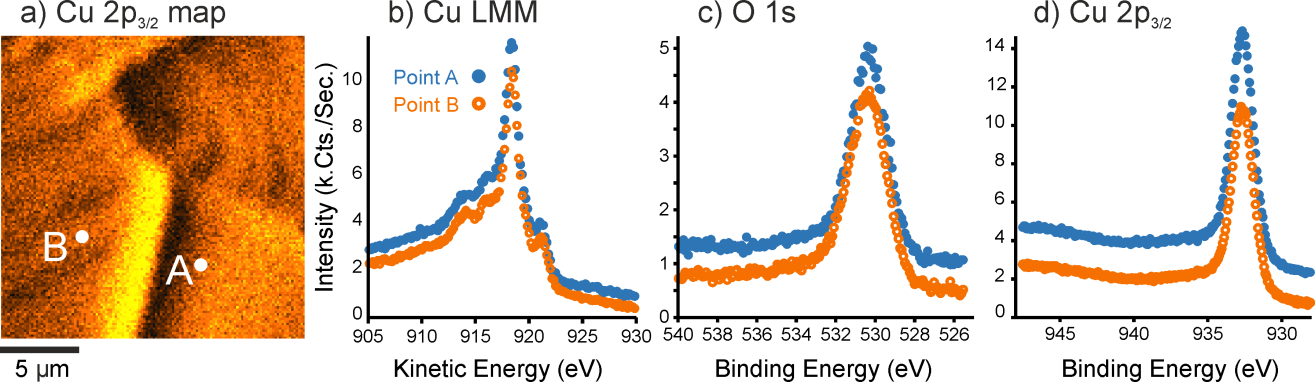


**Supplementary Figure 10:** Scanning photoemission microscopy image recorded under 4% O_2_ and 96% H_2_ at 650 °C. a) shows two grains, one with a flat (region A) and one with a stepped surface (region B). According to the Cu LMM spectra shown in b) both grains correspond to metallic Cu. The presence of oxygen as shown in the XPS spectrum in c) indicates that the surface is oxygen terminated, while the copper remains metallic (d). No quantitative difference in oxygen termination could be determined from our SPEM measurements.
