# Supplementary material for: In situ observation of oscillatory redox dynamics of copper
Source: Nat Commun. 2020 Jul 16;11:3554. doi: 10.1038/s41467-020-17346-7 (PMC7366672; doi:10.1038/s41467-020-17346-7)
Supplement: Supplementary file 3 — Description of Additional Supplementary Files [file 41467_2020_17346_MOESM3_ESM.pdf]

**Description of Additional Supplementary Files**

File Name: Supplementary Movie 1
Description: Cu annealing in $H_2$ at 700°C, 20 Pa.

File Name: Supplementary Movie 2
Description: Redox dynamics in region B at 650 °C in 95% $H_2$ and 5% $O_2$, 20 Pa.

File Name: Supplementary Movie 3
Description: Redox dynamics in region B at 400 °C in 94% $H_2$ and 6% $O_2$, 20 Pa

File Name: Supplementary Movie 4
Description: Oscillatory redox dynamics in region B at 700 °C in 96% $H_2$ and 4% $O_2$, 20 Pa. In this regime, different surface states including stepped and smooth morphologies as well as oxide growth and reduction occur sequentially. Wave-like propagation of boundaries between different surface phases is highlighted in the video by blue and red lines.

File Name: Supplementary Movie 5
Description: Oscillatory redox dynamics as in Supplementary Movie 4, but recorded at higher magnification.

File Name: Supplementary Movie 6
Description: Oscillatory redox dynamics on a 110 oriented single crystalline Cu foil at 700 °C in 96% $H_2$ and 4% $O_2$.
